# Aneurysmal bone cyst: results of an off label treatment with Denosumab

**DOI:** 10.1186/s12891-019-2855-y

**Published:** 2019-10-20

**Authors:** Hans Roland Dürr, Ferdinand Grahneis, Andrea Baur-Melnyk, Thomas Knösel, Christof Birkenmaier, Volkmar Jansson, Alexander Klein

**Affiliations:** 1 Orthopaedic Oncology, Department of Orthopaedics, Physical Medicine and Rehabilitation, University Hospital, LMU Munich, Marchioninistr. 15, D-81377 Munich, Germany; 2 Department of Radiology, University Hospital, LMU Munich, Munich, Germany; 3 Institute of Pathology, University Hospital, LMU Munich, Munich, Germany

**Keywords:** Aneurysmal bone cyst, Denosumab, Recurrence, Prognosis

## Abstract

**Background:**

The treatment of aneurysmal bone cysts (ABCs) has evolved and less invasive methods have been tried. Denosumab is a monoclonal antibody which inhibits osteoclasts. It has been shown to be effective in giant cell tumour of bone (GCT) of bone and hence promises some effect also in ABC. We report on 6 patients treated with Denosumab and compare our results to the cases already published.

**Methods:**

Data of 6 patients with ABCs and patients whose treatment included Denosumab were retrospectively analyzed. Denosumab was used at a dose of 120 mg on days 1, 8, 15 and 29, and every 4 weeks thereafter. In some of these patients the dose was reduced at the end of the treatment. Clinical and radiological responses were evaluated.

**Results:**

In 4 female and 2 male patients with a mean age of 17 years (range: 6–30 years) the lesions were located in the sacrum (2), in distal radius, distal femur, talus and pelvis. One of the sacral lesions healed after 12 months and has stayed stable for 3 years since. The second patient received 2 years of therapy with recalcification, but recurred 1 year later and is under renewed therapy. The pelvic lesion improved but recurred. This patient has a 13-years history of intermittent therapy including surgery, two pregnancies and remains in a stable situation. The lesion of the talus did not improve with Denosumab after surgery and was complicated by destruction of the ankle joint with osteoarthritis. Recurrent lesions of the distal femur and the distal radius, previously treated by curettage and bone grafting healed under Denosumab and have remained stable for 2 and 3 years, respectively. One case of severe hypercalcemia was observed in a 7-year old child 6 months after discontinuation of Denosumab.

**Conclusion:**

Denosumab provides a treatment option for ABCs in anatomically critical locations. Adjuvant application might reduce the rate of local recurrence. In young patients, severe rebound hypercalcemia months after discontinuation of Denosumab may occur.

## Background

Aneurysmal bone cysts (ABC) are considered benign yet locally aggressive lesions with a relevant potential for local recurrence. They typically appear in the metaphyses of the long bones and in the vertebral column and were first described by Jaffe and Liechtenstein in 1942 [1–3]. ABC’s are most often seen in children and young adults with no gender predilection. They are lytic, blood-filled, separated by fibrous septa and with histopathology typically showing fibroblasts, osteoclast-type giant cells and reactive woven bone [4].

ABC(s) were originally thought to be reactive in nature, caused by a circulatory abnormality leading to an increased venous pressure and resulting in dilation of the intraosseous vascular network [5, 6]. In 1999, Panoutsakopoulos et al. demonstrated a balanced chromosomal translocation t(16;17)(q22;p13) as a cytogenetic abnormality in primary aneurysmal bone cyst [7] involving the ubiquitin carboxyl-terminal hydrolase 6 (USP6) gene, located on chromosome 17p13. Since then, the neoplastic nature of ABC has been established and the USP6 translocation has since been found in approximately 75% of cases [8]. In differentiating primary ABC’s from secondary lesions or other tumors such as telangiectatic osteosarcoma this may be an option in selected cases. This particular translocation enhances the production of TRE17, a protease which leads to increased matrix metalloproteinase (MMP)-9 and increased MMP-10 activity [9]. This in turn is associated not only with blocking osteoblastic maturation via an autocrine mechanism involving bone morphogenetic dysregulation, but also increased release of VEGF (Vascular Endothelial Growth Factor) thus enhancing vascularization [10].

The treatment of ABC has changed over the years. Due to its often mutilating character, resection is not an acceptable option in most of the cases leaving intralesional procedures such as curettage as the standard of care [11]. Less invasive methods such as aggressive biopsy (“Curopsy”) [12], selective arterial embolization [13, 14], sclerotherapy with ethibloc or polidocanol [15] have been tried.

Denosumab is a human monoclonal antibody which binds specifically to the cytokine receptor activator of nuclear factor-kappa B ligand (RANKL) [16]. This prevents RANKL from activating the RANK receptor of osteoclasts, inhibiting osteoclast function. Denosumab is highly effective in giant cell tumour of bone (GCT) and therefore similar effects in principle could be hoped for in ABC, which has distinct similarities to GCT [17]. Up to now no protocol or treatment recommendation for the use of denosumab in ABC exists.

To our best knowledge, 2 case series (with 9 patients each) have previously been published [18–20] with an additional 11 cases having been published as individual case reports [20–29].

The aim of this study is to report our results from a series of 6 patients and to compare our experience to the data already published.

## Methods

Retrospectively all 65 patients with ABCs treated at our institution between 1982 and 2014 were analyzed with data having been collected in a prospective fashion. In 6 cases, Denosumab was used off-label in accordance to the established protocol in giant cell tumor (GCT) of bone [30]. The indications had been expected unreasonable morbidity of surgical treatment either in a primary or in a recurrent lesion or in an adjuvant setting after surgery for local recurrence. This study was approved by the ethics committee of our faculty (#18–373). Written consent was obtained from all patients included in this study. Denosumab was administered subcutaneously at a dose of 120 mg on days 1, 8, 15 and 29, and every 4 weeks afterwards. This scheme is later referred to as the so-called adult regimen. In a 6-year old boy, the dose was reduced as is described below. In some of the patients, the dose was reduced towards the end of the treatment as also described below. Calcium 500 mg and Vitamin D 1000 IU were supplemented on a daily basis. Clinical data was collected from the patients charts. Routine follow-up investigations such as magnetic resonance imaging (MRI) or radiographs and in specific cases also computed tomography (CT) were performed every 3 months in the first year of therapy, every 6 months in the second year and then based on individual decision. But as described in the literature ABCs should be considered as completely healed if recurrence does not occur within 2 years after the end of therapy [13].

## Results

From 2011 to 2018, 6 patients (4 female and 2 male) with histologically proven ABCs were treated with Denosumab. The mean age was 17 years (range: 6–30 years). Two lesions were located in the sacrum, one each in distal radius, distal femur, talus and pelvis. Pain was the leading symptom in all patients with a mean duration of 14 months (1–42 months) prior to diagnosis.

The two patients with sacral ABCs underwent needle biopsies prior to initiating treatment.

### Case 1

**Fig. 1:**
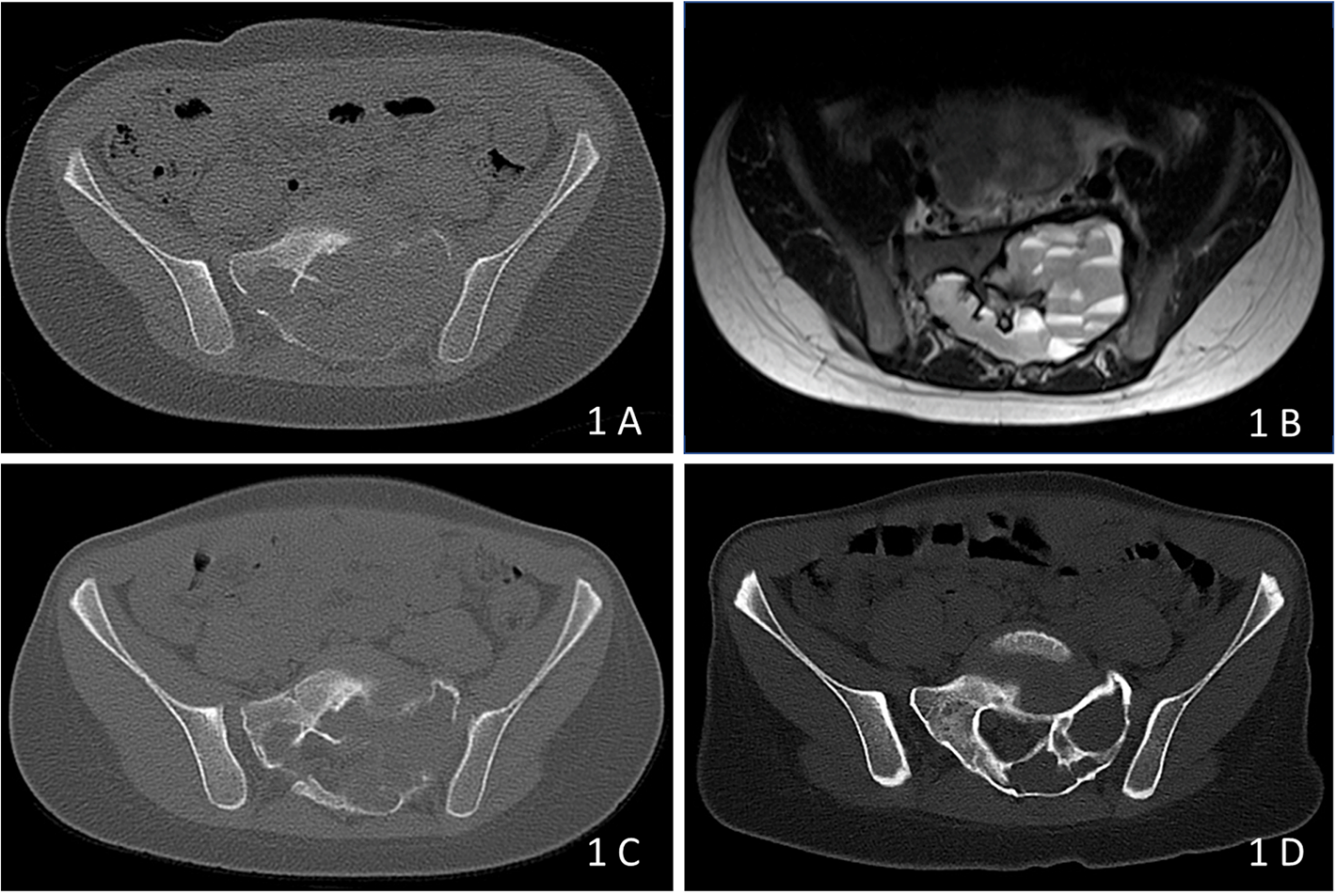
**a**, **b**: CT-scan (A) showing the massive destruction of the left and also in parts the right sacrum at the levels S1 and S2. The blood filling of the cyst is clearly visible as fluid-fluid levels in the T2-weighted MRI image (**b**). **c**: CT-scan 2 months after initiation of treatment showing increasing bone density at the margins of the lesion. **d**: CT-scan one after 1 year of treatment proofing the total bone restoration in the right and the impressive gain of bone at the left sacrum

In this 6-year-old boy, the Denosumab dosage regimen was adapted from a published trial of Densoumab in GCT by using 50% of the proposed adult dosage (60 mg every 4 weeks with two additional doses on days 8 and 15). Two months after the initiation of treatment the child was free of pain. A second CT-scan showed the ABC constant in size but with increasing bone density at the margins of the lesion (Fig. 1). At this point, the parents requested an additional embolization, but angiography showed no tumour vessels and no arterial irregularities and only a minimal embolization with 0.5 ml silicon spheres was performed. The Denosumab application was continued. After 1 year of treatment, the child was free of pain with normal growth and teething. The latest CT-scan showed an impressive growth of bone starting from the margins of the lesion with remnants of the cyst on the left and with complete filling of the defect on the right side. The treatment was stopped and a follow-up examination was scheduled for 12 months later. In October 2015, another CT scans showed nearly complete healing of the cyst. But as a consequence of Denosumab discontinuation, a severe rebound hypercalcemia had developed 6 months after the end of therapy and made intensive care treatment necessary.

### Case 2

An 18-year old male, also with a sacral ABC had an injection with polidocanol and an embolization not leading to any improvement. Three months later, Denosumab was initiated using the above mentioned adult regimen. After 1 year with clinical and radiological success, the dosage was gradually reduced and after 2 years Denosumab was stopped. One year later he developed a local recurrence as evidenced by MRI and Denosumab was restarted. 1 ½ year later with again radiologically confirmed recalcification the dosage was again reduced.

### Case 3

A 30-year old lady with a very large ABC of the left pelvis had curettage and bone grafting after CT-guided biopsy and developed a recurrence 1 year later. Again curettage and bone grafting was performed resulting in a second recurrence, 3 ½ year later. Denosumab was then initiated using the adult regimen. After 5 months, a clear response was documented, but after 8 months despite Denosumab, local recurrence was evident and due to the reduced stability around the acetabulum, curettage and bone grafting were again performed. Three months later, she became pregnant and 1 1/2 years after the previous surgery, another recurrence was seen and Denosumab was restarted after cessation of breast feeding 5 months after the diagnosis of LR. After 18 months, the situation was stable and the patient decided for a second pregnancy with cessation of Denosumab. Three years later and after an uncomplicated second pregnancy with twins, a recurrence was again obvious. A needle biopsy was performed that excluded GCT and confirmed ABC. Denosumab was started again resulting in partial sclerosing of the lesion. The dosage of Denosumab has since been reduced to 120 mg every 2 months.

### Case 4

In a 16-year old female after a second recurrence at the talus treated with bone grafting, Denosumab was used for 1 year with 120 mg per month leading to a stable situation. Two years later the lesion again recurred but now as a ganglioma due to the secondary destruction of the joint surface. In order to preserve all options for future treatment of the ankle joint, repeat curettage and bone grafting were performed.

### Case 5

**Fig. 2:**
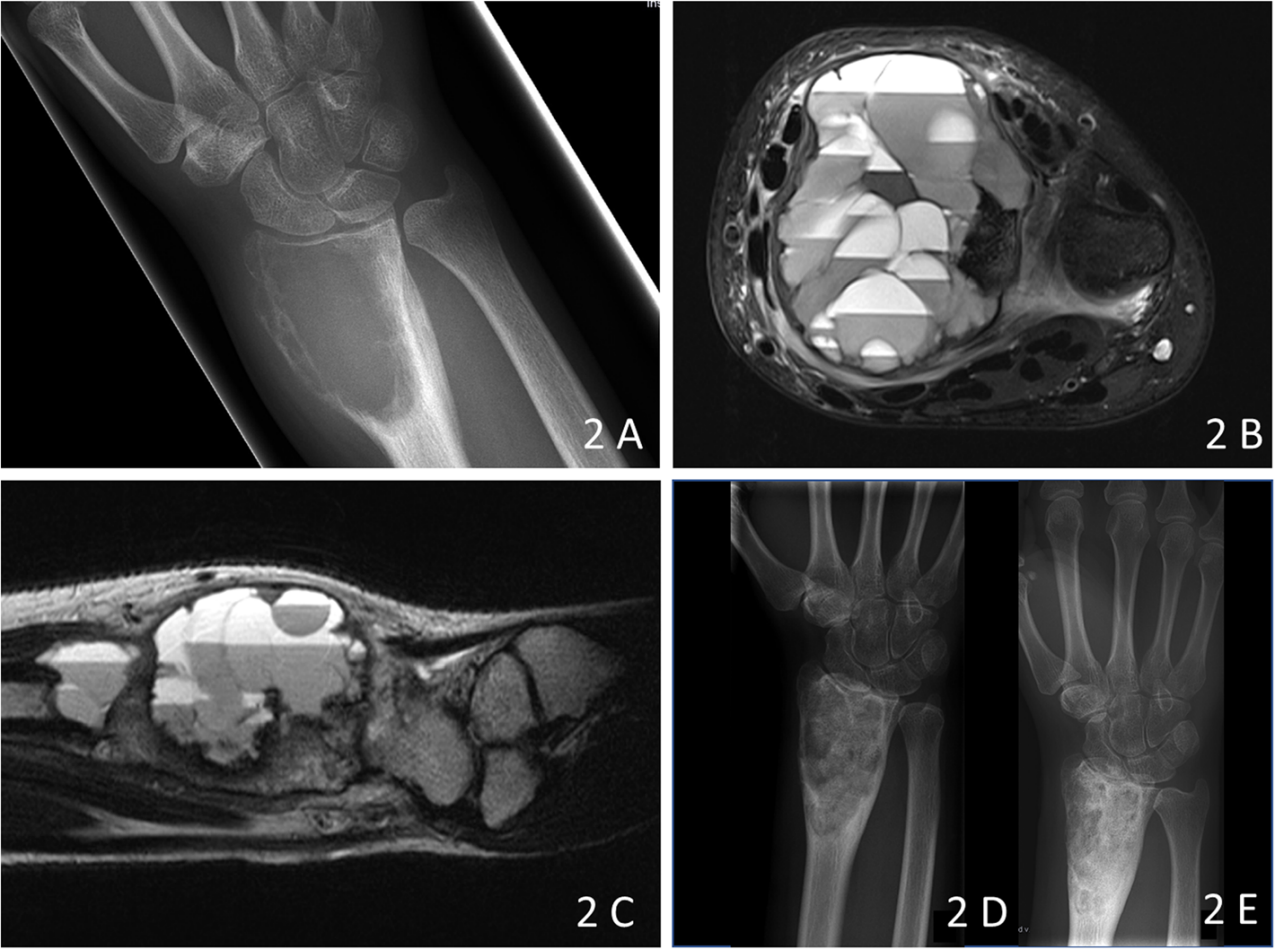
**a** Radiograph of the right distal radius showing the typical metaepiphyseal exentric osteolysis of an ABC of bone. **b**: The lesions shows typical fluid- fluid levels on T2-weighted TSE imaging with the patient in supine position. **c:** Local recurrence 3 months after curettage and bone grafting. **d**, **e**: Radiographs 2 months (**d**) and 1 year (**e**) after surgery and starting of Denosumab therapy

In a 15-year old female the lesion at the distal radius was treated with polidocanol injections and a simultaneous biopsy at the time of the first injection. Due to progression subsequently curettage and bone grafting were performed. Three months later due to recurrence the procedure had to be repeated (Fig. 2). Denosumab was then initiated in the adult regimen for 6 months leading to healing of the lesion with the latest follow-up now 3 years after that point.

### Case 6

In a 16-year old female after biopsy the lesion at the distal femur was treated with an embolization that had no effect and resulted in further growth of the cyst. Curettage and bone grafting were performed showing progressive local recurrence 3 and 5 months after surgery. Denosumab was begun according to the adult regimen for 1 year followed by 120 mg every 2 months for 6 months and by 120 mg every 3 months for another 6 months. Under this therapy, the lesion healed without any signs of recurrence and has been stable now for 2 years.

So in total Denosumab healed one sacral lesion and stabilized a second with recurrence after cessation and recalcification after re-establishment of therapy. The pelvic lesion improved but recurred after cessation of therapy. This particular patient has a 13-years history of intermittent treatment with two pregnancies in between and is currently in a stable situation. The lesion of the talus did not improve but was complicated by secondary osteoarthritic destruction. The recurrencies of the distal femoral and the distal radius lesion after curettage and bone grafting healed with Denosumab and have remained s for 2 and 3 years after cessation of therapy.

## Discussion

ABC are benign but locally aggressive lesions. Wide resection is not an option for the majority of patients because of the resulting disability. So intralesional curettage with or without bone grafting is still the predominantly used therapy, but carries a risk of local recurrence of about 20% [3]. Over the years, less invasive procedures have been propagated. Ethibloc, an alcoholic solution of a fibrogenic and thrombogenic agent proved to be effective with repeated injections but in some cases showed severe side effects [31]. In a randomized study with 94 patients, repeated injections of polidocanol showed better results than curettage alone (93% vs 85% healing) [32]. Repeated arterial embolizations have also been reported with success rates of more than 80% [13]. Also, repeated injections of doxycycline, an antibiotic with some potential to inhibit matrix metalloproteinases and angiogenesis was reported by Shiels et al. with a healing rate of 94% in their series [33]. An overview comparing the results of different forms of therapy including our own patients treated without Denosumab has recently been published [34].

In GCT the interaction of RANK and RANKL is an important factor which regulates the giant cell formation and progression of this tumor [35]. The pathophysiology of ABC seems to be similar to this [29, 36]. Denosumab, which effectively blocks interaction between RANKL and RANK has been approved for the treatment of osteoporosis, metastatic bone disease, multiple myeloma and GCT [19, 37]. The use of denosumab as an adjuvant treatment in patients with GCT has shown a high rate of recalcification [38].

In ABC the first report of Denosumab treatment dates back to 2012 [20]. Later 11 cases had been described in 9 studies. All patients showed regression of the lesion and recalcification. This treatment effect rendered surgery possible in two cases and the lesions were resected. The reported follow-up was rather inhomogenous with between 0 and 19 months after cessation of Denosumab. Local recurrence was described in just one case [21].

Kurucu et al. described 9 patients, 3 of them with surgical or non-surgical pretreatment [19]. Denosumab was given for 6–14 months with median 15 doses. All patients were free of symptoms after 3 months. Radiological improvement was evident in 8 cases. Follow-up time after the end of treatment was 10–24 months (median 15 months). 2 patients had further surgery, two other patients developed recurrence (after 16 months) or progressive disease and had renewed treatment with Denosumab or surgery. In total, the authors reported recurrence or progression in 4 of the 9 patients. In addition, they observed severe hypercalcemia in two patients 10 and 24 months after cessation of treatment.

Palmerini et al. reported 9 patients with Denosumab treatment [18]. It is not clear from their publication how many of the patients had any kind of prior therapy. Two patients had surgery after Denosumab. All 9 patients were classified as having sustained tumor control. At the last follow-up 5 patients were still on Denosumab treatment, 2 patients were disease-free after curettage and 2 patients are now 12 and 24 months without Denosumab and free of disease. No patient developed severe side effects.

Denosumab is effective in ABC as it is in GCT of bone. Our own results are similar to those described in both previously published case series. It is clear from those series as as well as from our own cases, that follow-up time after cessation of treatment is a major factor because recurrence, with some exceptions, needs time to develop. In this respect, the published cases may report better results than might be observed with longer follow-up. In general, all published cases demonstrated a clear clinical and radiological benefit of Denosumab in more than two thirds of the patients.

A major factor is the age of the patients. ABC is a lesion in young adults or children. The published series include patients as young as 2 years [19]. There is a risk of retardation in growth and disturbed dental developing with Denosumab not very well described up to now. The currently available knowledge is mainly based on individual treatment results in children suffering from fibrous dysplasia or osteogenesis imperfecta [39–43].

Most serious and as described also in one of our own patients is a rapid loss of the newly acquired bone caused by rebound formation and activation of osteoclasts once treatment is stopped, resulting in severe hypercalcemia [44, 45]. Other known adverse effects during denosumab therapy are hypocalcemia, necrosis of the jaw, fatigue, muscular pain or atypical femoral fractures if longer used [38].

## Conclusions

Denosumab provides an additional non-invasive method of treating ABCs in surgically critical locations such as the spine or the pelvis. As shown here, it may also reduce the rate of local recurrence with adjuvant application after intralesional surgery in aggressive lesions. Care providers need to be aware that the use of Denosumab in ABC is off-label and therefore requires a thorough interdisciplinary discussion with the patient or his/her family. The most common severe complication of Denosumab, osteonecrosis of the jaw, has not been described up to now in patients treated for ABC. But due to the low age of many of the children, severe rebound hypercalcemia months after cessation of therapy remains a considerable risk and implies that consequent laboratory follow-up for at least 2 years be performed.

## Data Availability

The datasets used and/or analyzed during the current study are available from the corresponding author on reasonable request.

## Abbreviations

ABC: Aneurysmal bone cyst
CT: Computed tomography
GCT: Giant cell tumour
IU: International Units
mg: Milligramms
MMP: Metalloproteinase
MRI: Magnetic resonance imaging
RANK: Cytokine receptor activator of nuclear factor-kappa B
RANKL: Cytokine receptor activator of nuclear factor-kappa B ligand
USP6: Ubiquitin carboxyl-terminal hydrolase 6
VEGF: Vascular endothelial growth factor
